# Hypofractionated External-Beam Radiotherapy for Prostate Cancer

**DOI:** 10.1155/2013/103547

**Published:** 2013-03-07

**Authors:** L. Chinsoo Cho, Robert Timmerman, Brian Kavanagh

**Affiliations:** ^1^University of Minnesota Medical Center, 420 Delaware Street SE, MMC-494, Minneapolis, MN 55455, USA; ^2^Department of Radiation Oncology, University of Texas Southwestern Medical Center at Dallas, 5801 Forest Park Road, Dallas, TX 75390, USA; ^3^Department of Radiation Oncology, University of Colorado School of Medicine, Anschutz Cancer Pavilion, Campus Mail Stop F-706, 1665 Aurora Court, Suite 1032, Aurora, CO 80045, USA

## Abstract

There are radiobiological rationales supporting hypofractionated radiotherapy for prostate cancer. The recent advancements in treatment planning and delivery allow sophisticated radiation treatments to take advantage of the differences in radiobiology of prostate cancer and the surrounding normal tissues. The preliminary results from clinical studies indicate that abbreviated fractionation programs can result in successful treatment of localized prostate cancer without escalation of late toxicity.

## 1. Introduction

Prostate cancer is the most common cancer diagnosed in American men after non-melanomatous skin cancer. According to the American Cancer Society estimate, there will be more than 241,000 new cases of prostate cancer in the United States in 2012. Approximately 28,000 men in the USA will die of prostate cancer, making it the second leading cause of cancer death in this country [1].

In most cases the prostate cancer is organ-confined at the time of initial diagnosis [2]. Radical prostatectomy and radiotherapy, either given as a seed implant or external beam radiation therapy, are the accepted standard options for treating the primary tumor itself, and androgen deprivation may be added selectively for certain cases with an intermediate or high risk of dissemination based on clinical and pathologic features evident at the time of diagnosis. Regarding the specific option of external beam radiotherapy, the current widely accepted standard regimen for organ-confined prostate cancer in the USA involves approximately eight weeks of fractionated treatments with a daily dose of 1.8–2.0 Gy to a total dose in the range of 70–80 Gy. At some centers the treatments, also called fractions, are given over 9-10 weeks [3]. 

Although many patients have been successfully treated with radiotherapy regimens of this nature, the optimal radiation schedule for the curative treatment of prostate cancer remains an unsettled question. For patients with clinical features suggesting at least an intermediate level of aggressiveness, a moderate dose escalation has been demonstrated to improve biochemical control with acceptable toxicity using contemporary radiotherapy techniques [4, 5]. Unfortunately, dose escalation using a conventionally fractionated treatment schedule requires a lengthened treatment course that is less convenient for patients and more costly for government and private insurance carriers. Emerging evidence accumulating from multiple recent studies indicates that more convenient and efficient shortened courses of radiotherapy for prostate cancer yield outcomes that are equivalent and possibly superior to the lengthier standard regimens. The scientific rationale for such “hypofractionated” treatment lies in the unique radiobiologic properties of prostate cancer. 

## 2. Radiobiologic Rationale

The radiobiological basis of hypofractionation for prostate cancer assumes that the prostate cancer cells respond to radiotherapy in a manner that can be mathematically modeled with a classic linear-quadratic equation:

(1) $$\begin{array}{c} S=S_{0}e^{-\alpha D-\;\beta D^{2}}, \end{array}$$

where *D* is the dose given, and *S* represents the cell survival after radiotherapy for initial cell population, *S*_*o*_. The constants, *α* and *β*, represent linear and quadratic components of the equation [6]. The equation may be rearranged to yield an estimate of the relative biological potency, called the biological effective dose (BED), of a fractionated course of therapy involving *n* individual treatments:

(2) $$\begin{array}{c} \text{BED}=nD\left(1+\frac{D}{\alpha /\beta }\right). \end{array}$$

The ratio *α*/*β*, which has units of Gy, characterizes the radiation sensitivity of a particular cell type. The *α*/*β* ratio is generally assumed to be approximately 10 Gy for most tumors and early-responding normal tissues and less than 5 Gy for late-responding tissues.

Conventional fractionation of 1.8–2.0 Gy/day is based on the premise that the therapeutic ratio, defined as the chance of eradicating tumor cells divided by the risk of normal tissue injury in late-responding normal tissue, is optimized by using small doses per fraction. The reason is that the tumor cell response, proportional to the BED as determined in the equation above, is generally less influenced by fraction size than is the BED for the late-responding normal tissues that surround the targeted tumor. However, in prostate cancer, tumor cells may have lower *α*/*β* than the surrounding normal tissues, and the opposite condition applies. The *α*/*β* ratio for prostate cancer is widely believed to be in the range 1–4 Gy [7–12]. In this context, it has been hypothesized that the therapeutic ratio would be enhanced by increasing the dose per fraction to the prostate cancer above the standard range [8–12]. 

It is noteworthy that the analyses that have yielded low *α*/*β* ratios for prostate cancer have sometimes involved comparisons of low dose rate brachytherapy with external beam radiotherapy, and assorted mathematical assumptions have been made [26–29] A comprehensive discussion of repair kinetics and other factors that influence the *α*/*β* estimates is beyond the scope of the present paper, and the reader is referred to some of the various studies related to this issue for additional discussion [10, 28, 30–35]. An added complexity is that it is not clear whether the traditional linear quadratic model continuing to model closely the radiation dose-response in tumors applies for fractional doses in the range of 6–8 Gy or higher [36–38]. Regardless, the aforementioned theoretical analyses, taken together, have informed the development of the hypothesis that there might be meaningful clinical advantage in the administration of radiation doses of greater than 2 Gy per fraction in the management of prostate cancer with external beam radiation therapy, and numerous clinical studies related to this hypothesis have been reported. 

## 3. Clinical Application of Hypofractionation for Prostate Cancer 

One of the early experiences using hypofractionation for prostate cancer came from Europe. Over 200 patients were treated at St. Thomas Hospital in London with hypofractionated radiotherapy to a dose of 55 Gy in 12 fractions and later to doses of 36 Gy in 6 fractions with low rectal and urological complications [39, 40]. Investigators of this retrospective review advocated 6 fractions in 3 weeks. Since the early reports of hypofractionation, there has been a steady increase in reports of hypofractionated radiotherapy for prostate cancer. Some have decreased the number of fractions modestly, and others have reduced it to only five sessions [41–50]. 

In the United States, Kupelian et al. [47] first reported their institutional experience using hypofractionation to treat safely an initial 100 consecutive patients with localized prostate cancer. Subsequently, an expanded experience involving 770 patients was reported [48]. All patients received intensity-modulated radiation therapy (IMRT) guided by daily prostate localization with a transabdominal ultrasound system. Patients were treated to total dose of 70 Gy in 28 daily fractional dose of 2.5 Gy. Biochemical failure, using both the ASTRO consensus definition [51] and the RTOG Phoenix definition (nadir+2 ng/mL) [52], was the study endpoint. The Radiation Therapy Oncology Group (RTOG) Morbidity System was used to assess treatment-related gastrointestinal (GI) and genitourinary (GU) morbidity. Fifty-one patients (51%) received androgen deprivation therapy for a period no longer than 6 months. The median followup was 66 months. The 5-year biochemical relapse free survival (bRFS) rates were 85% by the ASTRO Consensus definition and 88% by the RTOG Phoenix definition. Results were also reported according to prognostic groups. For low, intermediate and high-risk disease, the 5-year bRFS rates using ASTRO consensus definition were 97%, 88%, and 70%. The corresponding 5-year bRFS rates using the RTOG Phoenix definition were 97%, 93%, and 75%, respectively. The acute rectal toxicity scores were 0 in 20, 1 in 61, and 2 in 19 patients. A great majority of patients experienced grade 0-1 acute gastrointestinal (GI)/genitourinary (GU) toxicities. The actuarial late grade 3 rectal and urinary toxicity rate at 5 years was 3% and 1%, respectively. Routine implementation of IMRT with tight PTV margins and IGRT are likely to have contributed to reduce the treatment-related toxicities.

A recently completed RTOG 0415 Phase III trial assigned randomized patients to either the hypofractionation treatment strategy from the report by Kupelian et al. (70 Gy in 28 fractions) or to 73.8 Gy in 41 fractions of 1.8 Gy daily doses. Three-dimensional conformal radiotherapy and IMRT were allowed. The trial was restricted to those patients with low-risk prostate cancer (T1-2, and PSA <10 ng/mL, and Gleason score 2–6). Mature results of the study are not yet available.

A Canadian hypofractionated randomized trial has been reported by Lukka et al. [13] The trial compared a conventional dose of 66 Gy in 33 fractions, considered low by contemporary standards, to a hypofractionated regimen of 52.5 Gy in 20 fractions in men with low- and intermediate-risk prostate cancer. The dose per fraction was 2.625 Gy, slightly higher than the fractional dose used by Kupelian et al. CT based treatment planning was done but contemporary technique such as IMRT was not used. In this trial, the 5-year rate of failure (biochemical or clinical) was higher in the hypofractionated arm compared to the standard fractionation arm (60% versus 53%, *P* < 0.05). The inferior result seen with hypofractionated treatment may be explained by the fact that for any *α*/*β* ratio >0.2, the BED of 52.5 Gy in 20 fractions is expected to be lower than the BED of 66 Gy in 33 fractions. At a median followup of 5.7 years, there was no difference in 5-year actuarial rate of late grade 3 or higher GI/GU toxicity between the two arms. 

Pollack et al. [14] reported results of a hypofractionated trial in patients with intermediate to high-risk features. The fractional dose was 2.7 Gy per treatment. Intermediate risk was defined as total Gleason's score of 7, PSA between10–20 ng/mL, or ≥3 biopsy cores of combined Gleason's score ≥5, as long as no high-risk features were present. High risk was defined as Gleason's score 8–10, Gleason's score 7 in ≥4 cores, clinical T3 disease, or PSA >20 ng/mL. Up to 4 months of androgen-deprivation prior to randomization were permitted. However, it was discontinued after enrollment for patients with intermediate risk and continued for 2 years for those with high-risk. The clinical target volume (CTV) for intermediate-risk patients included the prostate and proximal seminal vesicles (approximately 9 mm). In high-risk patients, the CTV included at least 50% of the seminal vesicles, prostate, and any extraprostatic extension. In the high-risk patients, separate CTV and PTV were designed to treat the distal portions of the seminal vesicles, periprostatic, periseminal vesicle, external iliac, obturator, and internal iliac lymph nodes. Treatments were delivered using IMRT with hypofractionated doses to normal tissues calculated using an estimated *α*/*β* ratio of 1.5. The trial compared 76 Gy in conventional 2.0 Gy fractions to 70.2 Gy in 2.7 Gy fractions. The hypofractionated arm was estimated to be equivalent to 84.4 Gy in 2.0 Gy fractions and designed to be equivalent to 8 Gy increase over the standard fractionated total dose of 76 Gy. The 5-year result of the trial was recently reported [15]. There were 303 assessable patients entered between 2002 and 2006. There were 152 patients assigned to receive standard fractionation and 151 patients assigned to receive hypofractionation. Median followup was greater than 60 months in both arms. The rates of biochemical failure using the Nadir+2 ng/mL definition and clinical failure, consisting of either local-regional failure or distant metastasis (LRF/DM), and deaths without failure were reported. There were no statistically significant differences between the treatment arms in terms of biochemical failure, any failure, or late side effects. The 5-year rates of any failure for standard fractionation and hypofractionation were 14.4% and 13.9%, respectively. There were no statistically significant differences in GI toxicity between the arms. However, the GU toxicity was higher among patients who received hypofractionation at 5 years. Grade ≥2 gastrointestinal toxicities were seen in 5% and 6.8% of the conventional and hypofractionated groups, respectively, but transient genitourinary grade ≥2 toxicities occurred in 8.3% and 18.3%, respectively (*P* = 0.028), though they persisted in less than 10% at five years [60]. Factors associated with increased GU toxicity included pretreatment AUA symptoms score. Patients with AUA greater than 10 at baseline had increased risk for grade ≥2 toxicities, 11% versus 34%, respectively. Length of ADT did not influence GU toxicity. 

An Australian trial reported by Yeoh et al. compared a modest dose of 64 Gy in 32 treatments to hypofractionated arm of 55 Gy in 20 treatments in men with favorable-risk prostate cancer [16, 61]. The fractional dose in this trial was 2.75 Gy. Two hundred seventeen patients with T1-2 prostate carcinomas were randomized to either the standard or the hypofractionated arm between 1996 and 2006. Treatments were predominantly four-field box technique with customized blocks using 6–23 MV photons. At a median followup of 90 months, biochemical relapse-free survival (bRFS) was significantly better with hypofractionation when Phoenix definition was used (53% versus 34%, *P* < 0.5). However, there was no difference in bRFS rates when older ASTRO definition was used (44% versus 44%). Morbidity was measured with the LENT-SOMA questionnaires. Gastrointestinal and genitourinary toxicity did not differ significantly between fractionation schedules. Investigators found that the conventional fractionation independently predicted for worse biochemical failure and genitourinary symptoms at 4 years. 

Coote et al. [17] reported a British dose escalation study for prostate cancer using hypofractionated IMRT regimen using a higher dose per fraction. There were 60 patients with T2-3N0 M0 adenocarcinoma of prostate, and either Gleason's score ≥7 or PSA 20–50 ng/L. Patients received 57–60 Gy to the prostate in 19-20 fractions using five-field IMRT. All treatments were delivered with a fractional dose of 3 Gy, 5 days per week. The target volumes included prostate and seminal vesicle without incorporation of regional lymph nodes. All patients received neoadjuvant hormonal therapy for 3 months before radiotherapy to a maximum of 6 months. Toxicity was assessed 2 years post radiotherapy using the RTOG criteria, LENT/SOMA, and UCLA prostate index assessment tools. There was no acute RTOG grade 3 or 4 toxicity. At 2 years, there were 4% grade 2 GI and 4.25% grade 2 GU toxicity. There was no grade 3 or 4 GI toxicity but one patient developed grade 3 GU toxicity at 2 years. UCLA index data showed a slight improvement in urinary function at 2 years compared with pretreatment. LENT/SOMA assessments demonstrated worsening of bowel function at 2 years. Patients receiving 60 Gy were more likely to develop problems with bowel function than those receiving 57 Gy.

Martin et al. [18] reported a prospective Phase II trial also using 3 Gy per fraction to treat 92 patients between 2001 and 2004. Eligible patients had clinical stage T1c-T2c N0 M0 with Gleason's score ≥ 6 and various PSA levels. The study was designed to maintain a biologic equivalent rectal dose of 120 Gy_3_, assuming *a*/*β* ratio of 3 for late normal tissue effects. The treatments were delivered with 3 Gy per fraction, 5 days per week for 4 consecutive weeks to a minimum dose of 60 Gy to the CTV (entire prostate and the base of seminal vesicles). With a median follow-up of 38 months, severe acute toxicity (grade 3–4) was rare, occurring in only 1 patient. There was no ≥3 late toxicity. The rate of biochemical control was 97% at 14 months by the Phoenix definition and 76% at 3 years by the older ASTRO consensus definition. 

Recently, Dearnaley et al. [19] reported the preplanned interim safety analysis of randomized multicenter Conventional or Hypofractionated High-Dose Intensity-Modulated Radiotherapy for Prostate Cancer (CH HiP) trial. At the time of analysis, 444 patients with localized prostate cancer between 2002 and 2006 had been enrolled. The eligible patients had clinical T1b–T3a N0 M0 prostate cancer, PSA <30 ng/mL, Gleason' score ≤ 7, WHO performance status of 0-1, and estimated risk of lymph-node involvement <30%. Patients received androgen suppression for 3–6 months before and during radiotherapy, but it was optional for men with low-risk disease. Patients in the control group received 74 Gy in 37 fractions. Patients in the hypofractionated groups received either 60 Gy in 20 fractions or 57 Gy in 19 fractions. All treatments were 5 fractions per week. Treatment for both standard and hypofractionated was planned and delivered using an integrated simultaneous-boost technique with target volumes designed to deliver 80% of the total dose to the prostate and base or all seminal vesicles 96% to the prostate with 0.5–1 cm margin, and 100% to the prostate with a 0–0.5 cm margin. Regional lymph nodes were not included in the treatment target volumes. With median follow-up of 50.5 months, 4.3% in the standard fractionation (74 Gy) group had GI toxicity RTOG grade ≥2 at 2 years. The GI toxicity was lower in both hypofractionated treatment arms: 3.6% in the 60 Gy group and the 1.4% in the 57 Gy group. RTOG GU toxicity grade ≥2 were seen in 2.2% of patients in the 74 Gy group, 2.2% of patients in the 60 Gy group, and none in the 57 Gy group at 2 years. There were no statistically significant differences in cumulative incidence of side-effects between the groups. A future report will include mature biochemical control rates data.

A phase III trial from Italycompared the toxicity and efficacy of hypofractionated (62 Gy in 3.1 Gy daily doses, 4 times per week) versus conventional fractionation radiotherapy (80 Gy in 2 Gy daily doses, 5 times per week) in patients with high-risk prostate cancer [20]. 

One hundred sixty-eight patients were randomized to receive three-dimensional conformal radiotherapy to the prostate and seminal vesicles. All patients received a 9-month course of total androgen deprivation. The median follow-up was 32 and 35 months in the hypofractionation and conventional fractionation arms, respectively. No difference was found for late toxicity between the two treatment groups. There were 17% and 16% grade 2 GI toxicity and 14% and 11% GU toxicity at 3 years in the hypofractionation and conventional fractionation groups, respectively. The 3-year freedom from biochemical failure rates were 87% and 79% in the hypofractionation and conventional fractionation groups, respectively (*P* = 0.035). The authors concluded that with equivalent late toxicity between the two treatment groups, the hypofractionated treatment resulted in better PSA control. 

Other investigators have used similar 3 Gy per fractions in hypofractionated treatments. A randomized trial comparing the toxicity and efficacy of hypofractionated and conventionally fractionated external-beam radiotherapy from Lithuania was reported by Norkus et al. [21, 22] Forty-four patients in the standard treatment arm were irradiated with 74 Gy in 37 fractions, and 47 patients in the hypofractionated arm were given 13 fractions of 3 Gy with additional 4 fractions of 4.5 Gy to a total dose of 57 Gy. The clinical target volume includes the prostate and a base of seminal vesicles. There was no significant difference in PSA response during the first-year follow-up. No acute grade 3 or 4 toxicities were observed. The grade 2 GU acute toxicity was significantly lower in the hypofractionated arm: 19.1% versus 47.7% (*P* = 0.003). The median duration of overall GI acute toxicity was also shorter with hypofractionation: 3 versus 6 weeks (*P* = 0.017). The follow up is too short to draw any conclusion regarding this study.

Soete et al. [23] used even higher dose per fraction, 3.5 Gy, to treat 36 patients in a phase II trial. Patients were treated with 56 Gy in 16 fractions over 4 weeks. Acute toxicities were scored using the RTOG/EORTC criteria and the international prostate symptom index. None of the patients experienced grade 3-4 toxicity. The grade 2 GU and GI toxicities were in 44% and 36%, respectively. All GU and the majority of GI symptoms had resolved 2 months after treatment. Although no grade 3-4 side effects were observed, the investigators noted an increase of grade 1-2 early side effects as compared to a conventional regimen. 

Ritter et al. [24, 29] reported preliminary results of a multi-center phase I/II clinical trial that explored the increasingly hypofractionated radiation therapy for localized prostate cancer. The three increasing hypofractionated levels were 64.7 Gy in 22 fractions (2.94 Gy/fraction), 58.08 Gy in 16 fractions (3.63 Gy/fraction), and 51.6 Gy in 12 fractions (4.3 Gy/fraction). These regimens were designed to maintain equivalent predicted late toxicity of approximately 76 Gy in 38 fractions. When the tumor *α*/*β* of 1.5 Gy is assumed, the 2 Gy equivalent dose is estimated to be 82 Gy. Fractional doses were increased when acceptable acute and late toxicities were noted. All patients were treated with tomotherapy or linac based IMRT with daily image guidance. Three hundred seven patients with favorable to intermediate risk prostate cancer was accrued. Median follow-up for depending on the fractional dose level ranged from 16 to 42 months. Acute grade 2 GU symptoms occurred in 20–30% of patients. Four to 9% of patients experienced grade ≥2 GI symptoms during treatment, but it declined to 2% by 2 years. Actuarial rectal bleeding at 2 years did not differ significantly between fractional dose levels. The rate of rectal bleeding was 8%, but all resolved either spontaneously or with minor intervention. The 5-year, biochemical progression free survival (bPFS) for level 1 was 94.7%, with no difference between fractionation dose schedules (*P* = 0.95).

Menkarios et al. [25] treated 80 patients in a multi-institution phase I/II trial of three-dimensional conformal radiation therapy (3D-CRT) for favorable-risk group prostate cancer (T1a-T2a, Gleason ≤ 6 and PSA <10 ng/mL). The patients received 5 Gy weekly for a total dose of 45 Gy (5 Gy X 9). Primary end-points were feasibility and late GI toxicity by RTOG scale, while secondary end-points included acute GI toxicity, acute and late genitourinary (GU) toxicity, biochemical control, and survival. At a median follow-up of 33 months, there was no acute GU grade 4 toxicity. The rates of grade 1, 2, and 3 acute GU toxicities were 29%, 31%, and 5%, respectively. There was no acute GI grade 3 or 4 toxicity. Acute GI grade 1 and grade 2 toxicities were 30% and 14%, respectively. Cumulative late grade ≥3 GI toxicity at 3 years was 11%. The three-year actuarial biochemical control rate was 97%. Prospective trials of hypofractionations using greater than 5 fractions are summarized in Table 1.

## 4. Stereotactic Body Radiation Therapy for Prostate Cancer 

Stereotactic Body Radiation Therapy (SBRT) involves an ultra-abbreviated treatment regimen of 5 or fewer fractions administered using image guidance and precise treatment delivery techniques. SBRT has been established as safe and efficacious for early stage lung cancer and selected patients with oligometastatic cancer [62, 63], and it has also been explored in the treatment of prostate cancer [43–46, 50]. SBRT can be delivered safely using any of several commercially available treatment systems. In some cases the dose delivery involves a combination of multiple non-coplanar beams aimed at the target, and in other systems the delivery is accomplished with static intensity-modulated beams or rotating modulated arcs. The selective prospective trials of SBRT for prostate cancer is listed in Table 2. 

The first prospective trial of SBRT for prostate cancer was published by Madsen and colleagues [53, 54], who treated 40 patients with SBRT using a daily dose of 6.7 Gy to a total dose of 33.5 (6.7 Gy X 5) Gy. The fractionation schedule was calculated to be equivalent to 78 Gy in 2 Gy fractions using an estimated *α*/*β* ratio of 1.5. At the median follow-up of 41 months, there were no instances of grade 3 GI toxicity and only a single episode of acute grade 3 GU toxicity. There was no grade 3 or higher late toxicities. The PSA control rate was 90% by the Phoenix definition.

Tang et al. [55] used slightly higher dose per fraction (7 Gy) to treat 30 men a phase I/II study. The eligible men had low-risk prostate cancer and received 5 weekly dose of 7 Gy to a total dose of 35 Gy. The SBRT technique consisted of intensity-modulated radiotherapy (IMRT) with daily image guidance using implanted gold fiducials. All patients had at least 6 months of follow-up. The treatments were well tolerated and there was no grade 3 or 4 GI/GU toxicity. Although there were initial grade 2 toxicities (13% GU and 7% GI), these scores returned to or improved over baseline at 6 months. The biochemical control rate was not available at the time of initial reporting. 

King et al. [56, 57] reported a follow-up of a phase II trial. The treatment consisted of SBRT with a total dose of 36.25 Gy in 5 fractions using the Cyberknife treatment platform. There were 67 patients treated between 2003 and 2009. Eligible patients had low- to favorable-intermediate risk features, including PSA ≤10, total Gleason's score of 6 or 7, and clinical Stage T1c–T2b. At median follow-up of 2.7 years, there were no grade 4 toxicities. RTOG grade 3, 2, and 1 bladder toxicities were seen in 3% (2 patients), 5% (3 patients), and 23% (13 patients), respectively. Rectal grade 3, 2, and 1 toxicities were seen in 0, 2% (1 patient), and 12.5% (7 patients), respectively. The 4-year PSA relapse-free survival was 94%. 

Katz et al. [50, 64] reported an experience of SBRT treatment given to 304 patients with clinically localized prostate cancer. Most received 5 fractions of 7.25 Gy (total dose 36.25 Gy). At a median follow-up of 40 months (range, 9–58 months), 10 patients died of other causes and 9 were lost to follow-up. The 4-year actuarial freedom from biochemical failure is 98.5%, 93.0%, and 75%, for the low-, intermediate-, and high-risk groups. Late toxicity included 4.2% RTG grade 2 rectal, 7.8% grade 2 urinary, and 1.4% grade 3 urinary. Mean Expanded Prostate Cancer Index Composite (EPIC) score for urinary and bowel QOL declined at 1 month post-treatment and returned to baseline by 2 years. Mean EPIC sexual QOL declined by 23% at 1 month. Eighty percent of the patients potent at baseline remained potent at the time of recent analysis.

McBride et al. [58] reported a Phase I multi-institutional trial of SBRT, also using the CyberKnife delivery platform. Patients had National Comprehensive Cancer Network (NCCN)-defined, low-risk prostate adenocarcinoma (Gleason score, 2–6; clinical stage T1c-T2a; PSA ≤10 ng/mL). Eligible patients had prostate size ≤80 cc by ultrasound measurement and had American Urological Association (AUA) symptom scores ≤15. Thirty-four patients received 37.5 Gy delivered in 5 fractions (7.5 Gy per fraction), 9 patients received 36.25 Gy in 5 fractions (7.25 Gy per fraction), and 2 patients received other regimens. All treatments were completed within 10 days with a minimum of 12 hours between fractions. The planning target volume (PTV) was prostate only with 3–5 mm expansion. No patient received androgen deprivation. With the median follow-up of 44.5 months, none of the patients experience biochemical failure by Phoenix (nadir+2) definition. Thirteen patients experienced PSA bounces. The mean PSA bounce was 1.07 ng/mL. There was an episode of late grade 3 urinary obstruction requiring TURP, and there were 2 (5%) episodes of late grade 3 proctitis. SHIM, AUA, and EPIC scores were used to assess quality of life in 56% of the patients who filled out the questionnaires. In addition to the decrease in sexual function, there also was a small late decline in EPIC Bowel scores. However, there were no statistically significant changes in AUA scores or EPIC Urinary scores.

In addition to the progressively larger dose per fraction used in the previously mentioned clinical studies, researchers at University of Texas at Southwestern Medical Center at Dallas (UTSW) conducted an animal experiment using higher doses per fraction. Tumor bearing nude mice was given 15 Gy, 22.5 Gy, or 45 Gy in 3 weekly fractions. Only the 45 Gy group demonstrated sustained PSA and tumor volume decreases in most mice [65]. 

This preclinical data supported the clinical trial launched by Boike and colleagues at UTSW [59], who conducted a phase I study that escalated the total doses from 45 Gy to 50 Gy in 5 fractions. Eligible patients included those with prostate size ≤60 cm^3^, Gleason score ≤6 with PSA ≤20, Gleason's score of 7 with PSA ≤15, ≤T2b, and American Urological Association (AUA) score ≤15. The total dose levels were 45 Gy, 47.5 Gy, and 50 Gy in 5 fractions. All patients were treated with a minimum of 36 hours between fractions with no more than 3 fractions per week. At the time of the report, the median follow-up was 30 months, 18 months, and 12 months for the 45 Gy, 47.5 Gy, and 50 Gy groups, respectively. For all patients, GI grade ≥2 and grade ≥3 toxicity occurred in 18% and 2%, respectively, and GU grade ≥2 and grade ≥3 toxicity occurred in 31% and 4%, respectively. Rectal quality-of-life scores (EPIC) fell from baseline up to 12 months but trended back at 18 months. The AUA GU symptom score increases returned to baseline in the 45-Gy and 50-Gy group but persisted in the 47.5-Gy dose level patients. The 47.5-Gy dose level patients had significantly elevated AUA scores after treatment (*P* = 0.002) compared with those in other dose groups. In all patients, PSA control was 100% by the Phoenix definition. 

In light of the accumulating clinical evidence for hypofractionation, RTOG 0938 [66] has been initiated in the USA It is a prospective randomized phase II trial that will compare 36.25 Gy in 5 fractions of 7.25 Gy to 51.6 Gy in 12 fractions of 4.3 Gy. All treatments will be completed in 2.5 weeks.

## 5. Conclusion

There is a growing body of compelling evidence supporting the safety and efficacy of abbreviated radiotherapy schedules for prostate cancer. Especially provocative are the recent reports of contemporary clinical trials that utilized the latest planning, imaging, and delivery techniques. Many of these modern trials were designed with the traditional linear-quadratic response model of prostate cancer. Maturation of these trials and others in development will help to answer several key questions, including the confirmation of the expected improvement in the therapeutic ratio, long-term biochemical relapse free survival, and long-term quality-of-life parameters. In addition, the efficiencies gained by shortening the schedules of treatment afford an opportunity for improving patient convenience and reducing costs associated with radiotherapy for prostate cancer.

## Figures and Tables

**Table 1:** Prospective trials of hypofractionated external-beam radiotherapy (>5 fractions).

Author	No. of patients	Type of study	Patient characteristics NCCN risk group	HYPO FX total dose (Gy)/fractional dose (Gy)	STD FX total dose (Gy)/fractional dose (Gy)	Median follow up (months)	PSA control		Late GU toxicity			Late GI toxicity		
							Hypo	STD	Hypo	STD	Toxicity	Hypo	STD	Toxicity
Lukka et al. [13]	936	Phase III	Low-Intermediate risk	52.5/2.63	66/2	68	58%	62%*		NS			NS	

Pollack et al. [14, 15]	303	Phase III	Intermediate High risk	70.2 /2.7	76/2	>60 months			18.3%	8.3%	≥Gr-2	6.8%	5%	≥Gr-2
									*P* = 0.028			NS		

Yeoh et al. [16]	217	Phase III	Low risk	55 /2.75	64/2	90	53%	34%*		NS			NS	
							*P* < 0.05							

Coote et al. [17]	60	Phase I/II	T2-3 N0M0 and GS ≥7 or PSA 20–50	57–60/3	—	24	73%*	—	4% 4%	— —	Gr-2 Gr-3	9.5% 0%	— —	Gr-2 Gr-3

Martin et al. [18]	92	Phase II	T1c-2 CNXM0 Low-high risk	60/3	—	38	97%* at 14 months		3% 0%	— —	Gr-2 Gr-3	4% 0%	— —	Gr-2 Gr-3

Dearnaley et al. [19]	153	Phase III	T1B-3A N0M0 GS 6–8 PSA < 50 Low-high risk	57–60/3	74/2	50.5	—		4.82–9% 0.7–4.2%	3.5% 1.4%	Gr- 2 Gr-3	4.8–6.9% 0.7%	7.6% 0%	Gr-2 Gr-3
									NS			NS		

Arcangeli et al. [20]	168	Phase III	High Risk	62/3.1	80/2	32	87%	70%*	NS			NS		
							*P* = 0.035							

Norkus et al. [21, 22]	91	Phase III	T1-3 N0M0 GS ≤ 7 PSA ≤10	57/3–4.5	74/2	—	NS during 1st 12 months		—			—		

Soete et al. [23]	36	Phase II	T1-T3 N0M0	56/3.5	—	2	—		—			—		

Ritter et al. [24]	307	Phase I/II	Low-Intermediate risk	64.7/2.94 58.08/3.63 51.6/4.3	—	16–42	95%* at 5 years		3%	—	Gr-2	8.8%	—	Gr-2

									11%	—	Gr 2	4%	—	Gr- 2
Menkarios et al. [25]	81	Phase I/II	Low risk	45/5	—	33	97%*		0%	—	Gr-3	0%	—	Gr- 3
									4%	—	Gr-4	0%	—	Gr-4
									at 37 months			at 37 months		

No.: Number, HYPO FX: hypofractionation, STD FX: standard, Gy: Gray, FX: fractionation, *: by Phoenix definition, NS: not significantly different, GS: Gleason's score.

**Table 2:** Prospective trials of hypofractionated external-beam radiotherapy (5 fractions).

Author	No. of patients	Type of study	Patient characteristics NCCN risk group	HYPO FX Total dose(Gy)/fractional dose(Gy)	Median follow up (months)	PSA control	Late GU toxicity		Late GI toxicity	
Madsen et al. [53, 54]	40	Phase I/II	Low risk	33.5/6.7	60	93%*at 5 years	12.5% 2.5%	Gr-2 Gr-3	12.5% 0%	Gr-2 Gr-3

Tang et al. [55]	30	Phase I/II	Low risk	35/7	12	—	13% 0%	Gr-2 Gr-3	7% 0%	Gr-2 Gr-3

King et al. [56, 57]	67	Phase II	Low risk	36.25/7.25	32	94%*at 4 years	3% 5%	Gr-2 Gr-3	2% 0%	Gr-2 Gr-3

McBride et al. [58]	45	Phase I	Low risk	36.25/7.25 37.5/7.5	44.5	98%* at 3 years	17% 2%	Gr-2 Gr-3	7% 5%	Gr-2 Gr-3

Boike et al. [59]	45	Phase I	Low-Intermediate risk	45/9 47.5/9.5 50/10	12–30	100%*	9% 4%	Gr-2 Gr-3	4% 0% 2%	Gr-2 Gr-3 Gr-4
							Late toxicity		Late toxicity	
							after 90 days		after 90 days	

No.: Number, HYPO FX: hypofractionation, STD FX: standard, Gy: Gray, FX: fractionation, *: by Phoenix definition, NS: not significantly different, GS: Gleason's score.
